# Pediatric multiple sclerosis: a review

**DOI:** 10.1186/s12883-018-1026-3

**Published:** 2018-03-09

**Authors:** Raed Alroughani, Alexey Boyko

**Affiliations:** 1 Division of Neurology, Department of Medicine, Amiri Hospital, Arabian Gulf Street, 13041 Sharq, Kuwait; 2 Department of Neurology, Neurosurgery and Medical Genetic of the Pirogov’s Russian National Research Medical University and MS Clinic at the Usupov’s Hospital, Ostrovitianov str. 1, Moscow, 117997 Russia

**Keywords:** Pediatric multiple sclerosis, Multiple sclerosis, Clinically isolated syndrome, Acute disseminated encephalomyelitis, Neuromyelitis optics

## Abstract

**Background:**

Pediatric-onset multiple sclerosis (POMS) prevalence and incidence rates are increasing globally. No disease-modifying therapy are approved for MS pediatric population. Hence, we aim to review the literature on POMS to guide treating physicians on the current understanding of diagnosis and management of pediatric MS.

**Methods:**

The authors performed a literature search and reviewed the current understanding on risk factors and disease parameters in order to discuss the challenges in assessing and implementing diagnosis and therapy in clinical practice.

**Results:**

The revised International Pediatric MS group diagnostic criteria improved the accuracy of diagnosis. Identification of red flags and mimickers (e.g. acute disseminated encephalomyelitis and neuromyelitis optica) are vital before establishing a definitive diagnosis. Possible etiology and mechanisms including both environmental and genetic risk factors are highlighted. Pediatric MS patients tend to have active inflammatory disease course with a tendency to have brainstem / cerebellar presentations at onset. Due to efficient repair mechanisms at early life, pediatric MS patients tend to have longer time to reach EDSS 6 but reach it at earlier age. Although no therapeutic randomized clinical trials were conducted in pediatric cohorts, open-label multi-center studies reported efficacy and safety results with beta interferons, glatiramer acetate and natalizumab in similar adult cohorts. Several randomized clinical trials assessing the efficacy and safety of oral disease-modifying therapies are ongoing in pediatric MS patients.

**Conclusion:**

Pediatric MS has been increasingly recognized to have a more inflammatory course with frequent infratentorial presentations at onset, which would have important implications in the future management of pediatric cohorts while awaiting the results of ongoing clinical trials.

## Background

Multiple Sclerosis (MS), a chronic inflammatory autoimmune disease of the central nervous system (CNS), is most commonly diagnosed in (young) adults, but can also affect children. Pediatric MS, also referred to as pediatric-onset MS (POMS), early-onset MS or juvenile MS, is generally defined as MS with an onset before the age of 16 years (sometimes before the age of 18 years). Between 3 and 10% of patients with MS present under 16 years of age and < 1% under 10 years of age [1]. Pediatric MS has distinctive features and the disease course is different than in adults. Children are less likely to develop primary or secondary progressive MS in childhood. 98% of pediatric MS patients present with a relapsing–remitting (RR) course, compared with 84% of adult patients [2]. Relapses appear to be more frequent in patients with POMS compared with adult-onset MS [3].

Guidelines for pediatric MS recommend that treatment can be started early in the disease course [4, 5]. Disease-modifying therapies (DMTs) for adult patients with MS are also applied in pediatric MS. However, data from large pediatric cohorts are lacking and no large placebo-controlled studies have been published yet. Consequently, level 1 evidence for the appropriate treatment and its timing is still scarce.

## Methods

A group of neurologists with expertise in MS met as part of a scientific group (ParadigMS) to address the current understanding of pediatric MS, and to discuss the evolving research and ongoing therapeutic trials in pediatric population. Two MS experts (R.A., A.B.) performed a comprehensive literature search of MEDLINE, EMBASE, and Cochrane databases, systematically reviewing more than 80 published manuscripts from the last two decades that involved pediatric cohorts or any prospective or retrospective studies with at least 10 patients. Case studies with importance to pathology or which have clinical implications, were included as well. The expert panel met again to discuss the topic (pediatric MS) after being extensively reviewed by the authors (R.A., A.B.) and identified the relevant knowledge that needs to be presented in a review article to guide the treating physicians on diagnosis and management of pediatric MS patients.

## Results

### Clinical features

Children can present with a wide variety of manifestations including optic neuritis (ON), sensory, brainstem-cerebellar, and motor symptoms. The MS course in cases with onset at < 16 years of age is very similar in among populations from Italy, Russia, France, USA, and Kuwait [1–3, 6–10]. The clinical phenotype differs from that of adult patients, in that pediatric MS patients generally experience a more aggressive disease onset with disabling clinical symptoms [11], a polyfocal presentation at disease onset [12] and a higher relapse rate early in the disease course [13]. Though these findings are mostly from the USA and Europe, no were major regional differences in the epidemiological patterns or clinical features, meaning data outside of these regions are scarce. Overall, children tend to have a more favorable outcome after a first clinical event [13]. They also have slower disease progression over time: they take 10 years longer to reach secondary progressive disease phase compared to adults [2]. The relatively slow development of irreversible physical disability in children [14] is believed to result from better plasticity, allowing better recovery from relapses. In pediatric MS time from onset to confirmed disability may be relatively long, but disability milestone is reached at an earlier age.

Axonal damage occurs early in MS and contributes to the degree of clinical disability. In children with MS, there is more pronounced acute axonal damage in inflammatory demyelinating lesions than in adults [15]. Similar heightened axonal damage was observed in a case study of a 12-year-old patient [16]. Evidence was found in a study that was performed on archival biopsy and autopsy tissue of 19 children with demyelinating diseases: MS (*n* = 11) or clinically isolated syndrome (CIS) (*n* = 8). Median age at biopsy/autopsy was 13 years (range 4 – 17 years). The most important outcome was the significant increase, by 50%, of acute axonal damage in early active demyelinating lesions of pediatric patients (median = 1665 Amyloid Precursor Protein (APP) -positive axons/mm^2^) compared to adult patients (median = 1100 APP-positive axons/mm^2^, *p* = 0.0455). The numbers of APP-positive axons/mm^2^ were significantly higher in the prepubertal age group (< 11 years of age) compared to the pubertal age group (11–17 years, *p* = 0.0061) and adult patients (≥18 years, *p* = 0.0044). Furthermore, significantly more children showed multifocal MRI T2 lesions (71.4% vs 54.5%, *p* > 0.05). Also, the index lesion was larger in pediatric patients (81.8% vs 50% size > 2 cm). There was an increased inflammatory infiltration in pediatric MS lesions, which was shown to be associated with the extent of acute axonal damage in pediatric and adult patients (*r* = 0.5381, *p* = 0.0098).

Besides clinical features that are typical for demyelination, MS is associated with significant cognitive impairment in childhood. In a study in 63 patients, 19 (31%) fulfilled criteria for cognitive impairment [17]. Cognitive outcome in these patients can be heterogeneous, as cognitive performance deteriorated in 42 of 56 cases (75%) after 2 years of follow-up [18]. In a 5-year longitudinal study, cognitive impairment index deterioration was observed in 56% of patients, improvement in 25%, and stability in 18.8% [19].

### Prevalence and incidence

One of two methodological approaches to calculate the prevalence and incidence of pediatric MS are usually conducted in scientific publications, which could explain the variation in published data. Subtraction of POMS cases from the total MS cohort, or calculating an age-specific, population-based risk are the common methodological approaches. Other reasons for the variation in the prevalence and incidence are the use of different diagnostic criteria and of a different cut-off age among the studied pediatric cohorts, ranging from 15 to 18 years.

The worldwide prevalence and incidence of pediatric MS is unknown, but data from individual countries and MS centers are available (see Table 1). Several studies indicate that at least 5% of the total population with MS comprises of pediatric patients [6, 7]. Population studies and case-control series show that between 1.7% and 5.6% of the MS population is younger than 18 years of age [2, 6–8]. A recent study showed that incidence and prevalence of pediatric MS in Kuwait in 2013 were 2.1 and 6.0, respectively [20]. Incidence in general is highest in children between the age of 13 and 16.

**Table 1:** Incidence and prevalence of pediatric MS: results from various national cohorts

Country	Number	Age range	Syndrome	Diagnostic criteria	Prevalence	Incidence	Reference
Germany	126	≤ 15 years	MS	McDonald 2005		0.64	Reinhardt et al. [45]
Netherlands	86	< 18 years	ADS	Krupp 2007	–	0.66	Ketelsegers et al. [46]
UK	125	1-15 year	ADS	Krupp 2007	–	0.98	Absoud et al. [47]
Italy (Sardinia)	21	0-18 years	MS	Krupp 2013	26.92	2.85	Dell’Avvento et al. [48]
USA	81	0-18 years	ADS MS	Krupp 2007	–	1.66 0.51	Langer-Gould et al. [49]
Brazil	125	0-18 years	MS	Krupp 2007	5.5% of MS population		Fragoso et al. [50]
Iran (Shiraz)	88	1-18 years	ADS	–		0.19	Inaloo et al. [51]
Kuwait	122	< 18 years	MS	Krupp 2013	6.0	2.1	Alroughani et al. [20]

*ADS* acquired demyelinating syndromes

### Risk factors

There is a possible role for Epstein-Barr virus (EBV) in MS pathogenesis. This was suggested by the results of a multinational observational study, which included 137 pediatric MS patients from 17 sites across North- and South-America and Europe [11]. Non-MS controls were matched 1:1 by year of birth with an MS participant enrolled from the same region. The participants underwent standardized assays (ELISA) for IgG antibodies directed against EBV, cytomegalovirus, parvovirus B19, varicella zoster virus, and herpes simplex virus. Over 108 (86%) of children with MS, irrespective of geographical residence, were seropositive for remote EBV infection, compared to only 64% of matched controls (*p* = 0.025). The hazard ratio (HR) to be in the MS group in case of seropositivity for remote EBV was 2.8 (confidence interval (CI): 1.4 – 5.8) (*p* = 0.005). Only anti-EBV nuclear antigen titers were higher in the EBV-positive MS patients compared to EBV-positive non-MS controls (*p* = 0.003).

One of the main risk factors of MS, also confirmed in pediatric MS, is HLA DRB1*1501 [21]. DNA from 56 children with MS (< 16 years of age) was used for HLA-DRB1 typing and compared to healthy controls (*n* = 328), MS patients from the same population (*n* = 234), and 76 parents of 39 pediatric MS patients. Only the frequencies of DR2(15) alleles were higher in both sets of MS patients than in controls. Transmission disequilibrium test (TDT) results showed significant difference in transmission of DR2(15) and non-DR2(15) alleles (*p* = 0.00002).

### Natural history

High-quality studies of the natural history of pediatric MS are scarce due to methodological issues. In a comparative study analyzing data collected from two different pediatric cohorts, clinical characteristics and progression of pediatric onset MS (< 16 years) over time were assessed. In the Moscow cohort, 67 cases of newly diagnosed MS were prospectively observed for 2 - 13 years, while the Vancouver cohort consisted of 116 MS cases who were retrospectively observed for 1 - 47 years [1, 9]. The pediatric cohorts were compared with an historical adult cohort to assess the risk of disability progression assessed by expanded disability status scale (EDSS) scores. There were a number of significant differences between pediatric and adult-onset MS [10]. The 50% risks to reach EDSS 3 and 6 were 23 and 28 years after MS onset, compared to 10 and 18 years in the comparator group.

In a longitudinal prospective population-based study the risk of disease progression in POMS was assessed [22]. The interval to second relapse was longer in pediatric patients (5.0 vs 2.6 years, *p* = 0.04) PPMS was less common (0.9% vs 8.5%, *p* = 0.003). Pediatric patients took longer to develop secondary progressive MS (SPMS) (32 vs 18 years, *p* = 0.0001) and to reach disability milestones (EDSS 4.0, 23.8 vs 15.5 years, *p* < 0.0001; EDSS 6.0, 30.8 vs 20.4 years, *p* < 0.0001; EDSS 8.0, 44.7 vs 39 years, *p* = 0.02), but did so between 7.0 and 12 years younger than in adult-onset MS. A high relapse rate predicted faster progression. Complete recovery on the other hand, reduced the risk of progression (reaching EDSS 4) on the long term.

#### Risk of conversion

Children with initial CIS are more likely to develop MS than those with acute disseminated encephalomyelitis (ADEM) as initial diagnosis. In a study of 123 children (< 18 years of age) with a combined retrospective and prospective follow-up (median 61.5 months), conversion from CIS to MS occurred in 26 of 67 children (38.8%); from ADEM to MS in 4 of 47 children (8.5%) [23]. Female gender, brain stem or hemispheric involvement, and Callen’s magnetic resonance imaging criteria [24] were found to predict the diagnosis of MS. Cerebrospinal fluid (CSF) did not prove to be a good indicator for conversion.

A second relapse and initial presentation with brain stem, cerebellar or cerebral dysfunction, or multifocal CIS were strongly associated with the development of MS (*p* = 0.002) in a retrospective study [25]. Sixteen patients (50%) experienced a second demyelinating event, with a mean interval between the first and second episode 21 (± 20) months. 11 (34%) developed pediatric MS after a mean follow-up of 6.1 (± 1.6) years. Asymptomatic brain lesions on MRI and the presence of oligoclonal bands were not predictors of conversion to MS in this study.

#### MRI parameters

MRI parameters can also be used to predict the risk of MS in children with CIS. In a national prospective inception cohort study at 23 sites in Canada, 284 eligible participants (age < 16 years) were followed up for 3.9 years [26]. Fifty-seven (20%) were diagnosed with MS after a median of 188 days. The presence of either one or more T1-weighted hypointense lesions (HR 20.6) or one or more periventricular lesions (3.34) was associated with an increased likelihood of MS diagnosis. This risk was particularly elevated when both parameters were present (HR 34.27).

A meta-analysis of 14 studies that included children presented with optic neuritis, revealed that older children and those with brain MRI abnormalities at presentation are at greater risk for MS [27]. Data of 223 patients (age range: 2 - 17.8 years) were analyzed. For every 1-year increase in age, the odds of developing MS increased by 32% (odds ratio (OR) = 1.3, *p* = 0.005). The risk of MS was greater in children with abnormal brain MRI scans at presentation compared with normal MRIs (OR = 28.0, *p* < 0.001).

## Discussion

### Prognosis

In a large cohort from a network of French and Belgian centers, patients with pediatric MS reached secondary-progression and disability milestones at ages approximately 10 years younger than patients with adult-onset disease, despite a slower development of irreversible disability [2]. Among the 17,934 patients, 394 (2.2%) had MS starting at 16 years of age or younger, and 290 (73.6%) of these patients were women. The mean age at onset was 13.7 years. Onset occurred at the age of 14 years or younger in 159 patients (40.4%), and at 10 years or younger in 30 patients (7.6%). The estimated median time between the first two neurologic episodes was 2.0 years.

A more aggressive disease course may be predicted by relapse severity and residual disability in early pediatric MS. In a retrospective study of 105 patients with MS or CIS onset prior to 18 years of age, optic nerve involvement was associated with a severe initial demyelinating event (IDE) (OR 4.30, *p* = 0.007) [28]. A severe initial demyelinating event was associated with incomplete recovery (OR 6.90, *p* < 0.001), with similar trends for second and third events. Incomplete recovery from the first event predicted incomplete second event recovery (OR 3.36, *p* = 0.055).

The importance of presentation at onset for the prognosis is underlined by a study of prognostic indicators of SPMS in a cohort of 127 pediatric MS patients (< 18 years of age) from Kuwait [29]. Twenty patients (15.8%) developed SPMS. At MS onset, brainstem involvement (adjusted HR 5.71; *p* = 0.010) and age at MS onset (adjusted HR 1.38; *p* = 0.042) were significantly associated with the risk of SPMS.

### Diagnostic criteria

Many different diagnostic criteria for pediatric MS have been proposed. It is challenging to rule out other disorders that may mimic MS, and to distinguish pediatric MS from various demyelinating syndromes that can occur in childhood. The criteria by the Pediatric International Study Group have been applied in most studies. This is because they have classified the various acquired demyelinating syndromes (ADSs) that may be the first clinical sign of pediatric MS. The classification of ADSs, which dates from 2007 [30] and has been updated in 2013 [31] is as follows (see Table 2 for diagnostic criteria):

- Pediatric MS
- Optic neuritis (ON)
- Transverse myelitis (TM)
- Clinically isolated syndrome (CIS)
- Neuromyelitis Optics (NMO)
- Acute disseminated encephalomyelitis (ADEM)

**Table 2:** Diagnostic criteria for pediatric MS [31]

For the diagnosis pediatric CIS, all of the following is required: − A monofocal or polyfocal, clinical CNS event with presumed inflammatory demyelinating cause. − Absence of a prior clinical history of CNS demyelinating disease (e.g. absence of past optic neuritis (ON), transverse myelitis (TM) and hemispheric or brain-stem related syndromes). − No encephalopathy (i.e. no alteration in consciousness or behavior) that cannot be explained by fever. − The diagnosis of MS based on baseline MRI features (as recently defined) are not met.
For pediatric ADEM, all of the following is required: − A first polyfocal, clinical CNS event with presumed inflammatory demyelinating cause. − Encephalopathy that cannot be explained by fever. − No new clinical and MRI findings emerge 3 months or more after the onset. − Brain MRI is abnormal during the acute (three-month) phase. − Typically on a brain MRI: • diffuse, poorly demarcated, large (> 1–2 cm) lesions involving predominantly cerebral white matter; • deep grey matter lesions (e.g. thalamus or basal ganglia) may be present; • T1-hypointense lesions in the white matter are rare.
For pediatric NMO, all of the following are required − Optic neuritis. − Acute myelitis. − At least two of three supportive criteria: * contiguous spinal cord MRI lesion extending over three vertebral segments; * brain MRI not meeting diagnostic criteria for MS; * aquaporin IgG seropositive status.
For pediatric MS, one of the following is required − ≥ 2 non-encephalopathic, clinical CNS events with presumed inflammatory cause, separated − by > 30 days and involving more than one CNS area. − One non-encephalopathic episode typical of MS which is associated with MRI findings consistent with 2010 Revised McDonald criteria for dissemination in space (DIS) and in which a follow-up MRI shows at least one new enhancing or non-enhancing lesion consistent with dissemination in time (DIT) MS criteria. − One ADEM attack followed by a non-encephalopathic clinical event, three or more months after symptom onset, that is associated with new MRI lesions that fulfill 2010 Revised McDonald DIS criteria. − A first, single, acute event (e.g. a CIS) that does not meet ADEM criteria and whose MRI findings are consistent with the 2010 revised McDonald Criteria for DIS and DIT (applied only to children ≥12 years old).

There are a number of important changes when comparing the 2007 and 2012 definitions for pediatric acute demyelinating disorders of the CNS [30, 31].

- Arguably the most important change is in the definition of MS (and pediatric MS). “Multiple clinical episodes of CNS demyelination separated in time and space” in the 2007 criteria, has been specified to “≥ 2 non-encephalopathic clinical CNS events with presumed inflammatory cause, separated by > 30 days and involving more than one CNS area”.
- Added to the definition of NMO has been the following condition: “Brain MRI not meeting diagnostic criteria for MS”.
- Encephalopathy is defined as “An alteration in consciousness (e.g. stupor, lethargy) or behavioral change unexplained by fever, systemic illness or post-ictal symptoms”.
- A second event is “The development of new symptoms at least three months after the incident illness irrespective of steroid use”.
- Multiphasic ADEM is defined as two episodes consistent with ADEM separated by 3 months but not followed by any further events. The second ADEM event can involve either new or a re-emergence of prior neurologic symptoms, signs and MRI findings.
- Relapsing disease following ADEM that occurs beyond a second encephalopathic event is no longer consistent with multiphasic ADEM, but rather indicates a chronic disorder, most often leading to the diagnosis of MS or NMO.
- Children with MS (under age 12) differ clinically from adolescents with MS. They are more likely than adolescent-onset MS patients to have an ADEM-like first attack, they can have large, ill-defined lesions early in the disease course, and they are less likely to have CSF oligoclonal bands.

### Differential diagnosis

As in adults, dissemination in time and space is an essential feature. In general, the more atypical the case and the younger the child, the more consideration is necessary before making a diagnosis of MS [32]. MS must not only be differentiated from acute ADEM or NMO, but there is also an extensive list of other disorders that can mimic MS which need to be excluded. Examples of such disorders are systemic lupus erythematosus (SLE), neurosarcoidosis, Sjögren syndrome, leukodystrophies, hereditary metabolic disorders, and encephalitic or meningo-encephalitic infectious etiologies.

It is especially challenging to determine whether a child with an initial demyelinating event (IDE) will develop subsequent events that are consistent with MS or not. A list of “red flags” in the differential diagnosis in children presented with their initial demyelinating event have been suggested [32]. It includes encephalopathy and fever, progression from the onset, involvement of the peripheral nervous system or other organs, absence of CSF oligoclonal IgG (40-50% of pediatric MS patients exhibit oligoclonal bands, which is less than in adults) and markedly elevated CSF white blood cells or proteins.

ADEM typically presents as a monophasic demyelinating disease. It may be induced by preceding viral infections or vaccination, e.g. concerning measles or varicella zoster virus (VZV). Seizures or behavioral disorders as common presenting symptoms in ADEM. It can be difficult to differentiate ADEM from the first MS attack based on clinical evaluation. MRI appearance plays a major role in the diagnosis. Two or more periventricular lesions, absence of a diffuse bilateral lesion pattern, and the presence of black holes are frequently seen in MS patients compared to patients with ADEM [33].

The appearance of new lesions in different locations on follow-up MRI strongly suggests MS. Recently it was shown that susceptibility-weight imaging (SWI) may be useful in differentiating initial presentation of pediatric MS from ADEM [34].

### Disease-modifying therapies

Studies in adult MS patients suggest significant benefit of early institution of DMTs. The available efficacy data for pediatric MS patients is scarce and mostly based on retrospective studies. An international consensus highlighted the importance of initiating DMT in children and adolescents with MS [35]. A rationale for early institution of DMT in pediatric MS patients was supported by several facts related to natural history data:

- 85-90% has an active relapsing MS course.
- Relapse rate is high in initial phases of the disease and is correlated with a bad prognosis.
- Short duration between relapses and the subsequent accumulation of disability.
- Although progression may be slower than in adults, moderate-to-severe disability is reached at a younger age.
- Brain tissue shows more active inflammation in childhood, so patients may benefit from the anti-inflammatory effects of DMTs.
- Despite the apparent clinical recovery from relapses due to better neuronal plasticity, cognitive impairment is frequent. Postponing treatment may have a negative impact on social activities and school performance.

Evidence of the effectiveness of DMTs in reducing relapse rate and disease progression in pediatric MS patients is exclusively based on observational studies. Four randomized controlled trials are either recruiting or reaching final stages: PARADIGMS (fingolimod), TERIKIDS (teriflunomide), FOCUS (dimethyl fumarate) and CONNECT (dimethyl fumarate vs. Interferon beta 1a).

### First-line treatment

The current first-line treatment of MS in children consists of either interferon beta (IFNB) or glatiramer acetate (GA). The safety profile of IFNB / GA remains favourable in children. No unexpected adverse events and no serious adverse events were documented in 44 pediatric MS patients from 7 countries who were treated with interferon beta-1b [36]. The mean age at the start of therapy was 13 years; 8 patients were ≤ 10 years of age. Most common adverse events included flu-like syndrome (35%), abnormal liver function test (26%), and injection site reaction (21%).

Adult doses of subcutaneous (sc) IFNB-1a (44 and 22 μg, three times weekly) were generally well tolerated by children and adolescents, with no new or unexpected adverse drug reactions, in a large retrospective study, named REPLAY [37]. It is the largest multicenter, multinational review of safety, tolerability, and efficacy outcomes with sc IFNB-1a in pediatric MS patients. Reviewed were records of 307 patients aged between 2 and 17 years, who had received at least 1 injection of sc IFNB-1a for demyelinating events. Despite the lack of a control group, beneficial effects were observed. Annualized relapse rates were 1.79 before and 0.47 during treatment. On the other hand, the experience with glatiramer acetate is very limited. It has roughly the same efficacy as IFNB [38].

### Second-line treatment

In children with breakthrough disease (defined as relapses while on first-line therapies), escalation to higher efficacious second-line therapies, such as natalizumab, fingolimod, mitoxantrone, cyclophosphamide, rituximab, and daclizumab may be considered based on the extrapolated data from adult cohorts. However, data on the safety, efficacy, and tolerability of most of these treatments are scarce and have been reported only in small-size retrospective case series [40].

Large observational studies have shown that natalizumab is an effective treatment in children with breakthrough disease, with a good safety and efficacy profile, comparable to those in adult populations [39–44]. A strong suppression of disease activity was observed in all subjects during follow-up in a study of 19 patients (mean age 14.6 +/− 2.2 years) [41]. The mean EDSS score decreased from 2.6 ± 1.0 to 1.9 ± 1.0 (*p* < 0.001). EDSS remained stable in 5 cases, decreased by ≥0.5 point in 6 cases, and decreased by ≥ 1 point in 8 cases. There were no relapses during follow-up (*p* < 0.001), nor new gadolinium-enhanced (Gd+) lesions (*p* = 0.008). A study by the same group of 55 patients showed a dramatic decrease in the number of relapses [42]. The mean number of relapses before treatment was high: 4.4. During follow-up only 3 relapses in all occurred. Mean EDSS scores decreased from 2.7 to 1.9 at the last visit (*p* < 0.001). During follow-up, the majority of patients remained free from MRI activity. Transient and mild clinical adverse events occurred in 20 patients. Anti-JCV antibodies were detected in 20 of 51 tested patients. In a retrospective study of 9 pediatric patients with highly active MS, the use of natalizumab completely halted the inflammatory process [43]. Two patients still had relapses, but they both had neutralizing antibodies against natalizumab. The median EDSS score decreased from 3.0 to 1.0, the median ARR decreased from 3.0 to 0.0. A recent study revealed that treatment with natalizumab was associated with reductions in mean ARR (3.7 vs 0.4; *p* < 0.001), n median EDSS scores (2 vs 1; *p* < 0.02), and in mean number of new T2-lesions per year (7.8 vs 0.5; *p* < 0.001) in children with active relapsing MS.

## Conclusion

Pediatric MS has long been an underdiagnosed and undertreated condition. It has distinctive features and the disease course is different than in adult-onset MS. Progression may be slower than in adults due to neuroplasticity, but moderate-to-severe disability is reached at a younger age. It is important to limit the axonal damage secondary to extensive inflammatory changes seen earlier in the disease process by initiating early DMT in these patients and delaying disability accumulation. More prospective, randomized, large cohort studies are needed to assess the safety and efficacy of DMTs in children with MS, especially in those with highly active disease or an aggressive disease course.

## Abbreviations

ADEM: Acute disseminated encephalomyelitis
APP: Amyloid Precursor Protein
CFS: Cerebrospinal fluid
CI: Confidence interval
CIS: Clinically isolated syndrome
CNS: Central nervous system
DIS: Dissemination in space
DIT: Dissemination in time
DMT: Disease-modifying therapies
EBV: Epstein-Barr virus
EDSS: Expanded disability status scale
GA: Glatiramer acetate
Gd+: Gadolinium-enhanced
HR: Hazard ratio
IDE: Initial demyelinating event
IFNB: Interferon beta
NMO: Neuromyelitis Optics
MRI: Magnetic resonance imaging
MS: Multiple sclerosis
POMS: Pediatric-onset MS
PPMS: Primary progressive MS
ON: Optic neuritis
OR: Odds ratio
RRMS: Relapse remitting MS
SC: Subcutaneous
SLE: Systemic lupus erythematosus
SPMS: Secondary progressive MS
SWI: Susceptibility-weight imaging
TDT: Transmission disequilibrium test
TM: Transverse myelitis
VZV: Varicella zoster virus
